# Parenthood and neurosurgery in Europe, a white paper from the European association of neurosurgical societies’ diversity in neurosurgery committee, part II – practice with children

**DOI:** 10.1016/j.bas.2023.102717

**Published:** 2023-12-08

**Authors:** Claudia Janz, Uri Pinchas Hadelsberg, Marike Broekman, Claudio Cavallo, Doortje Engel, Gökce Hatipoglu Majernik, Anke Hoellig, Tijana Ilic, Hanne-Rinck Jeltema, Dorothee Mielke, Ana Rodríguez-Hernández, Yu-Mi Ryang, Saeed Fozia, Nikolaos Syrmos, Kristel Vanchaze, Pia Vayssiere, Silvia Hernandez-Duran

**Affiliations:** a Department of Neurosurgery, Hôpitaux Universitaires de Genève (HUG), Geneva, Switzerland; b Faculty of Medicine, Université de Genève (UNIGE), Geneva, Switzerland; c Dept of Neurosurgery, Haaglanden Medical Center, The Hague and Dept of Neurosurgery, Leiden University Medical Centre, Leiden, the Netherlands; d Department of Neurosurgery, Neurocenter of Southern Switzerland, Switzerland; e Regional Hospital of Lugano, Lugano, Switzerland; f Department of Neurosurgery, Canton Hospital, St. Gall, Switzerland; g Department of Neurosurgery, Hadassah University Medical Center, Jerusalem, Israel; h Department of Clinical Neurological Sciences, Schulich School of Medicine and Dentistry, Western University, London, Ontario, Canada; i Department of Neurosurgery, University Hospital RWTH Aachen, Germany; j National Department of Neurosurgery, Centre Hospitalier de Luxembourg, the Netherlands; k Städtisches Klinikum Solingen, Neurochirurgische Klinik, Gotenstrasse 1, 42563, Solingen, Germany; l Department of Neurosurgery, University Medical Center Groningen, Groningen, the Netherlands; m Department of Neurosurgery, University Hospital Göttingen, Göttingen, Germany; n Dept. of Neurological Surgery, Germans Trias i Pujol University Hospital, Universidad Autónoma, Barcelona, Spain; o Dept. of Neurosurgery &Center for Spinetherapy, Helios Klinikum Berlin-Buch, Germany; p Department of Neurosurgery at Leeds General Infirmary, Leeds, United Kingdom; q Aristotle Univesrity of Thessaloniki, Greece; r AZ St Lucas, Ghent and AZ Alma, Eeklo, Belgium; s Klinik für Neurochirurgie, Universitätsmedizin Göttingen, Robert-Koch-Straße 40, 37075, Göttingen, Germany

**Keywords:** Parental leave, LGBTQ+, Childcare, Breastfeeding, Single parenting

## Abstract

**Introduction:**

In the first part of this White Paper, the European Association of Neurosurgical Societies (EANS) Diversity in Neurosurgery Committee (DC) addressed the obstacles faced by neurosurgeons when planning to have a family and practice during pregnancy, attempting to enumerate potential, easily implementable solutions for departments to be more family-friendly and retain as well as foster talent of parent-neurosurgeons, regardless of their gender identity and/or sexual orientation. Attrition avoidance amongst parent-neurosurgeons is at the heart of these papers.

**Research question:**

In this second part, we address the obstacles posed by practice with children and measures to mitigate attrition rates among parent-neurosurgeons. For the methodology employed to compose this White Paper, please refer to Supplementary Electronic Materials (SEM) 1.

**Materials and methods:**

For composing these white papers, the European Association of Neurosurgical Societies (EANS)'s Diversity Committee (DC) recruited neurosurgeon volunteers from all member countries, including parents, aspiring parents, and individuals without any desire to have a family to create a diverse and representative working group (WG).

**Results:**

In spite of the prevailing heterogeneity in policies across the continent, common difficulties can be identified for both mothers and fathers considering the utilization of parental leave.

**Discussion and conclusion:**

Reconciliation of family and a neurosurgical career is challenging, especially for single parents. However, institutional support in form of childcare facilities and/or providers, guaranteed lactation breaks and rooms, flexible schedule models including telemedicine, and clear communication of policies can improve working conditions for parent-neurosurgeons, avoid their attrition, and foster family-friendly work environments.

## Highlights

• While the provision of a gender-neutral family leave would enable parents from both sexes to accommodate their schedules around their family life, and also provide LGBTQ + parents with the possibility to take equal leave, major European legislation would be necessary to be able to implement this measure.
• Challenges of going back to practice do exist, with the following being clear examples:
  o Discrimination related to motherhood is a common concern reported by female neurosurgeons on a survey in the United States
  o Sleep deprivation due to newborn sleep-wake schedule in combination with on-call duties.
• As a neurosurgeon, shift-work, on-call duties, and unpredictable working hours are commonplace. Thus, returning to practice presupposes accommodating a busy work schedule into family life and ensuring that children are cared for while the parent neurosurgeon resumes surgical duties.
• Breastfeeding provides short-term and long-term health and economic and environmental advantages to children, women, and society. Not breastfeeding, however, is associated with lower intelligence and economic losses of about $302 billion annually or 0·49% of world gross national income
• The committee suggests an array of solutions.
• Single-parent problems, logistical challenges and career stagnation are presented as well.

## 1 Introduction

In the first part of this White Paper, the European Association of Neurosurgical Societies (EANS) Diversity in Neurosurgery Committee (DC) addressed the obstacles faced by neurosurgeons when planning to have a family and practice during pregnancy, attempting to enumerate potential, easily implementable solutions for departments to be more family-friendly and retain as well as foster talent of parent-neurosurgeons, regardless of their gender identity and/or sexual orientation. Attrition avoidance amongst parent-neurosurgeons is at the heart of these papers. In this second part, we address the obstacles posed by practice with children and measures to mitigate attrition rates among parent-neurosurgeons. For the methodology employed to compose this White Paper, please refer to Supplementary Electronic Materials (SEM) 1 (see Table 1, Table 2).

**Table 1:** European countries where joint legal parenthood for same-sex couples is possible and associated parental leave policies.

Joint legal parenthood for same-sex couples possible	Austria
	Belgium
	Denmark
	Estonia
	Finland
	France
	Germany
	Ireland
	Italy
	Luxembourg
	Malta
	The Netherlands
	Portugal
	Spain
	Sweden
	Slovenia
	United Kingdom
Joint legal parenthood for same-sex couples NOT possible	Bulgaria
	Croatia
	Czechia
	Cyprus
	Greece
	Hungary
	Latvia
	Lithuania
	Poland
	Romania
	Slovakia
All types of partners of parents can take parental leave	Austria
	Belgium
	Estonia
	Finland
	France
	Germany
	Ireland
	Luxembourg
	The Netherlands
	United Kingdom
Only legal parents can take parental leave	Bulgaria
	Czechia
	Greece
	Italy
	Latvia
	Lithuania
	Portugal
	Romania
	Spain
Only married or registered partners can take parental leave	Hungary

**Table 2:** Parental leave policies in Europe.

Country	Paid maternity leave (weeks)	Payment (percentage of salary)	Paid paternity leave (weeks)	Payment (percentage of salary)	Paid parental leave to mothers (weeks)	Payment (percentage of salary)	Paid parental leave for fathers (weeks)	Payment (percentage of salary)
Austria	16	100	0	0	44	76	9	76
Belgium	15	64	2	73	17	20	17	20
Bulgaria	59	90	2	90	52	33	0	0
Croatia	30	100	0	0	26	42	0	0
Cyprus	18	75	2	75	0	0	0	0
Czech Republic	28	61	1	61	35	84	1	61
Denmark	18	53	2	53	32	53	0	0
Estonia	20	100	2	100	146	44	0	0
Finland	18	74	3	63	144	19	6	63
France	16	90	2	90	26	14	26	14
Germany	14	100	0	0	44	65	9	65
Greece	43	50	1	100	0	0	0	0
Hungary	24	70	1	100	136	38	0	0
Ireland	26	27	2	27	0	0	0	0
Israel	15	100	0	0	0	0	0	0
Italy	22	80	1	100	26	30	0	0
Latvia	16	80	1	100	78	50	0	0
Lithuania	18	100	4	100	44	100	0	0
Luxembourg	20	100	2	100	17	67	17	67
Malta	18	86	1	100	0	0	0	0
Netherlands	16	100	1	100	0	0	0	0
Norway	13	94	0	0	78	40	10	94
Poland	20	100	2	100	32	68	0	0
Portugal	6	100	5	100	24	60	17	44
Romania	18	85	1	100	91	85	4	85
Slovakia	34	75	0	0	130	21	0	0
Slovenia	15	100	4	90	37	90	0	0
Spain	16	100	4	100	0	0	0	0
Sweden	13	78	1	59	43	57	13	78
Switzerland	14	80	0	0	0	0	0	0
Turkey	16	67	1	100	0	0	0	0
UK	39	30	2	19	0	0	0	0

Definitions: Maternity leave (or pregnancy leave): employment-protected leave of absence for employed women directly around the time of childbirth (or, in some countries, adoption). Paternity leave: employment-protected leave of absence for employed fathers at or in the first few months after childbirth. Paternity leave is not stipulated by international convention. Parental leave: employment-protected leave of absence for employed parents, which is often supplementary to specific maternity and paternity leave periods, and frequently, but not in all supplementary to specific maternity and paternity leave periods, and frequently, but not in all countries, follows the period of maternity leave.

### 1.1 Parental leave utilization

The first part of this paper summarized the parental leave policies of different European countries. They can be accessed separately as SEM 2. In general terms, parental leave is defined as an employment-protected leave of absence for employed parents. It is often supplementary to specific maternity and paternity leave periods. Duration, conditions, and payment differ even more widely between the European countries (Database et al., 2019). In spite of the prevailing heterogeneity in policies across the continent, common difficulties can be identified for both mothers and fathers considering the utilization of parental leave.

### 1.2 Mothers

• Maternity leave, defined as the employment-protected leave of absence for employed women directly around the time of childbirth (or, in some countries, adoption) varies greatly between countries, ranging from only 6 weeks in Portugal to 43 in Greece and even 59 in Bulgaria. Eligibility criteria for payment vary between the countries and may depend on the contract duration, the time woman have been working in the months preceding birth, as well as the prior insurance contributions. Payment ranges from 27% in Ireland to 100% in several countries and is usually based on the prior earnings with or without a ceiling. In Ireland, however, payment is fixed on 245 Euro per week (Database et al., 2019).
  o Financial concerns may discourage mothers from utilizing maternal leave, as payment is not always equivalent to the salary they would normally earn, in addition to new costs incurred in due to the newborn child.

### 1.3 Fathers

• Paternity leave is not stipulated by international convention and in general, periods are much shorter as compared to maternity leave. However, taking into account the short length, workers on paternity leave often receive full wage payments (Database et al., 2019). Nevertheless, depending on the country of practice, fathers utilizing paternity or parental leave can be subject to financial disadvantages.
  o Fathers, especially in same-sex families, are subject to great disadvantages when desiring to spend time with their newborn children, as leave for men is less than for women.
  o A “stalled revolution”, society not keeping pace with women entering the workforce, has taken place, also to the detriment of men desiring to share the more traditionally female roles in the household (Casper and Bianchi, 2009).
• Absentee fathers have been shown to negatively impact the development of their children.
  o In a 2015 paper by Cools et al. from Norway (Cools et al., 2015), a four-week period of paternal leave created a more equal distribution of children-related tasks between the mother and father. Furthermore, it also was able to show that it improved children's exam scores at the end of compulsory schooling.

### 1.4 LGBTQ + community

• As mentioned in the first part of this paper, members of the LGBTQ + community do not have parenthood rights in some European countries, and their access to parental leave is not comparable to that of heterosexual couples (Picken and Janta, 2019).

### 1.5 All

• A common concern amongst parent-trainees is “loss of time”. The utilization of parental leave presupposes delaying graduation date from their neurosurgical training, while also pausing their technical skills development.
• Fear of “missing out” on surgical experience and being “left behind”.

### 1.6 Potential solutions

• While the provision of a gender-neutral family leave would enable parents from both sexes to accommodate their schedules around their family life, and also provide LGBTQ + parents with the possibility to take equal leave, major European legislation would be necessary to be able to implement this measure.
  o Department chairs can negotiate individually with their center's management the provision of gender-neutral family leave and communicate it openly to their team.
• Guaranteed continuation of training and/or employment and career advancement after parental leave, preferably in the form of a standard of practice (SOP) within each unit, delineating what new parent-neurosurgeons can expect when returning to practice and how they can resume their surgical and clinical activities.
• Access to skills laboratories and simulators can provide new parent-neurosurgeons with the opportunity to continue honing their surgical prowess while on parental leave.

### 1.7 Challenges of going back to practice

• Discrimination related to motherhood is a common concern reported by female neurosurgeons on a survey in the United States (Gupta et al., 2021).
  o Coming back after parental leave therefore means having to convince your superiors that you are able to fulfill their expectations, albeit cultural norms between geographic regions influencing the variability of female surgeons' experiences (Xepoleas et al., 2020).
• Sleep deprivation due to newborn sleep-wake schedule in combination with on-call duties.
  o Sleep deprivation in surgeons impacts cognitive performance and puts their own and patients' health at risk (Parker and Parker, 2017). Cognitive performance seems to be more impacted than technical performance (Banfi et al., 2019), (Whelehan et al., 2020).
  o While the risks of a “sleepy surgeon” were recognized through the implementation of the European Working Time Directive (2003/88/EC) (Working Conditions), limiting the number of working hours during the week, the number of continued on-call hours and enforcing rest periods after 24h shifts, no accommodations have been made for sleep-deprived parents.

### 1.8 Childcare availability

As a neurosurgeon, shift-work, on-call duties, and unpredictable working hours are commonplace. Thus, returning to practice presupposes accommodating a busy work schedule into family life and ensuring that children are cared for while the parent neurosurgeon resumes surgical duties.

• Limited formal childcare options have been shown to have negative consequences for career development, especially in women (Carlson et al., 2021).
• On a survey among surgical residents from the US, childcare support was offered through the residency program or hospital in only 18.4%. Most often, this was in the form of preferential daycare enrollment, discounted daycare, or access to backup childcare services, but 85.9% of women who were surveyed reported that these options did not offer hours that accommodated a surgical resident's schedule. A total of 260 residents (75.4%) reported that additional childcare support would have helped them focus on their surgical training (Rangel et al., 2018).
• In Europe, all member countries operate policies that reduce the cost of non-parental childcare for parents. However, Legal entitlements, which grant parents guaranteed access to childcare, vary across the EU, and most of these entitlements are for the duration of school hours (Rastrigina et al., 2020).
  o Childcare coverage is insufficient to accommodate a neurosurgeon's schedule and duties.

### 1.9 Breastfeeding

• Breastfeeding provides short-term and long-term health and economic and environmental advantages to children, women, and society. Not breastfeeding, however, is associated with lower intelligence and economic losses of about $302 billion annually or 0·49% of world gross national income (Rollins et al., 2016), (Victora et al., 2016).
• Despite these benefits, the American Academy of Pediatrics and the World Health Organization have noted that the work environment, in both policy and structure, is often not supportive of women who choose to breastfeed. A paper by Barber-Madden (Barber-Madden et al., 1987) identified insufficiently comprehensive maternity leave policies, lack of child care at or near the workplace, rigid time schedules that do not allow for nursing breaks, lack of a location providing privacy for breast-pumping, and no facilities for refrigeration of pumped breastmilk as barriers to breastfeeding at the workplace. Mothers in neurosurgical professions are equally concerned by these issues, with the additional problem of fitting breastfeeding with surgical schedules.

### 1.10 Potential solutions

• Provision of napping/resting areas not only for the staff on-call, but also for parents with young children, so that they can rest in between cases.
• Schedule accommodation with shorter shifts/schedules the day before a challenging surgical case to allow the parent-surgeon extra time to sleep/”rest-banking” (Business).
• Telemedicine, a concept proven to work during the Covid-19 pandemic in neurosurgery (Eichberg et al., 2020), could be extended to accommodate parent-neurosurgeons to do outpatient clinic and research remotely, as to be able to spend more time with their children while still fulfilling their clinical duties.
• On-site 24h childcare facilities/providers would enable the surgeon to perform surgeries at all hours of the day while their child is taken care of near them; alternatively, on-call childcare providers for surgeons on-call could offer the same coverage and enable surgeon full employment. Specifically, in big tertiary facilities with neurosurgery departments, these centers should consider in-house childcare facilities on the premises.
• Establishment of lactation facilities within the departments, where new mothers can breastfeed, pump, refrigerate pumped breastmilk (Business).
• Regarding work load during parenthood- Perhaps a shift from longer, more complicated cases, which were done when the resident did not have any kids, to shorter, less time-demanding cases should be done for parents. Albeit controversial, one may propose operating on less time-demanding cases in the latter part of residency, if needed, after having kids.
• Guaranteed provision of nursing/pumping breaks during long surgeries (Business).

### 1.11 Being a single parent

Reconciliation of family and career is one of the major challenges of our time, and this holds especially true for single parents. In the United States, the proportion of children living in married families of two biological partners has declined over time (Brown, 2010). About 23% of children resided in single-mother families in 2009 versus 11% in 1970. In Europe, there is also an increasing trend of children living in single-parent households over the past decade. Furthermore, single parenthood continues to be heavily gendered, with 11% of the families in 2019 being led by single mothers, as opposed to 3% by single fathers (Nieuwenhuis, 2020).

### 1.12 Emotional distress

• Employed single mothers reported higher levels of fatigue in parenting than do non-employed partnered mothers (Meier et al., 2016).
• Among college-educated women with family, those with a career spend a larger share of their day unhappy, sad, stressed and tired (Bertrand, 2013).
• Although single-parent families hold on to an expectation of a positive experience of togetherness, they are typically left with a feeling of disillusionment and guilt, stating that “there is never enough, one is at the service of children” (Daly, 2001).

### 1.13 Logistical challenges

• While some EU countries prioritize their childcare facility allocation based on household composition in favor of single parents (Rastrigina et al., 2020), coverage during long working schedules is still not available.
• As a single parent, relying on a partner to make the schedules fit is not possible (Nieuwenhuis, 2020), (Carroll, 2017).
• Being a single parent has been shown to have a detrimental impact on the parent's financial security and wellbeing, partly due to the extra costs of childcare (Buddeberg-Fischer et al., 2010; Kahn et al., 2014; Aisenbrey et al., 2009).

### 1.14 Career stagnation

• Being the main childcare provider presupposes little time available for career-advancing measures, such as research, visiting external training seminars or medical conferences, and attending training courses.
• In some countries, completing your training program will make it necessary to work in different departments all over the country and to move house regularly. This is already a major problem for partnered mothers but especially endangers completion of the residency rotation in single mothers.

## 2 Conclusions

Reconciliation of family and a neurosurgical career is challenging, especially for single parents. However, institutional support in form of childcare facilities and/or providers, guaranteed lactation breaks and rooms, flexible schedule models including telemedicine, and clear communication of policies can improve working conditions for parent-neurosurgeons, avoid their attrition, and foster family-friendly work environments.

## Declaration of competing interest

All the authors who have participated in the drafting, writing and submitting this manuscript report no conflict of interests pertaining to this paper.

## Appendix A Supplementary data

Multimedia component 1 Multimedia component
1
