# Supplementary material for: Parenthood and neurosurgery in Europe, a white paper from the European association of neurosurgical societies’ diversity in neurosurgery committee, part II – practice with children
Source: Brain Spine. 2023 Dec 8;4:102717. doi: 10.1016/j.bas.2023.102717 (PMC10951702; doi:10.1016/j.bas.2023.102717)
Supplement: Multimedia component 1 [file mmc1.docx]

**Methodology**

For composing these white papers, the European Association of Neurosurgical Societies (EANS)’s Diversity Committee (DC) recruited neurosurgeon volunteers from all member countries, including parents, aspiring parents, and individuals without any desire to have a family to create a diverse and representative working group (WG).

Meetings were organized via the videoconference platform Zoom® (Zoom Video Communications, Inc. California, USA) on a bimonthly basis for a one-year period. During the initial phase of the project, meetings were carried out on a biweekly basis and different aspects of parenthood were discussed, taking into consideration the personal experiences of the WG members. During these initial discussions, obstacles and challenges in practicing neurosurgery while becoming and/or being a parent were recognized.

In a second stage, literature pertaining to the obstacles and challenges identified was collected. For the literature search, PubMed and Google Scholar were utilized to identify scientific papers pertaining to parenthood in medicine. Furthermore, European agencies were digitally consulted to clearly define the legal framework within which parents and aspiring parents are to practice neurosurgery, including parental leave policies and parental leave rights in Europe. This information was then summarized and compiled as a GoogleDoc (Google LLC California, USA).

Once literature was collected and sorted according to the pertaining problems identified, WG members discussed potential, implementable solutions to tackle points discussed. Then, a second round of literature searches was conducted to identify evidence supporting potential solutions. To ensure objective interpretation and collection of data, every member of the WG reviewed the literature identified, following the principles of horizontal organizations.

Finally, manuscripts were drafted and reviewed by all the members of the WG.
